# Transposable Element Genomic Fissuring in *Pyrenophora teres* Is Associated With Genome Expansion and Dynamics of Host–Pathogen Genetic Interactions

**DOI:** 10.3389/fgene.2018.00130

**Published:** 2018-04-18

**Authors:** Robert A. Syme, Anke Martin, Nathan A. Wyatt, Julie A. Lawrence, Mariano J. Muria-Gonzalez, Timothy L. Friesen, Simon R. Ellwood

**Affiliations:** ^1^Centre for Crop and Disease Management, Department of Environment and Agriculture, Curtin University, Bentley, WA, Australia; ^2^Centre for Crop Health, University of Southern Queensland, Toowoomba, QLD, Australia; ^3^Department of Plant Pathology, North Dakota State University, Fargo, ND, United States; ^4^Cereal Crops Research Unit, Red River Valley Agricultural Research Center, Agricultural Research Service, United States Department of Agriculture, Fargo, ND, United States

**Keywords:** barley net blotch, *Hordeum vulgare*, single molecule sequencing DNA, optical mapping, synteny, transposable element, repeat annotation

## Abstract

*Pyrenophora teres*, *P. teres* f. *teres* (PTT) and *P. teres* f. *maculata* (PTM) cause significant diseases in barley, but little is known about the large-scale genomic differences that may distinguish the two forms. Comprehensive genome assemblies were constructed from long DNA reads, optical and genetic maps. As repeat masking in fungal genomes influences the final gene annotations, an accurate and reproducible pipeline was developed to ensure comparability between isolates. The genomes of the two forms are highly collinear, each composed of 12 chromosomes. Genome evolution in *P. teres* is characterized by genome fissuring through the insertion and expansion of transposable elements (TEs), a process that isolates blocks of genic sequence. The phenomenon is particularly pronounced in PTT, which has a larger, more repetitive genome than PTM and more recent transposon activity measured by the frequency and size of genome fissures. PTT has a longer cultivated host association and, notably, a greater range of host–pathogen genetic interactions compared to other *Pyrenophora* spp., a property which associates better with genome size than pathogen lifestyle. The two forms possess similar complements of TE families with Tc1/Mariner and LINE-like Tad-1 elements more abundant in PTT. Tad-1 was only detectable as vestigial fragments in PTM and, within the forms, differences in genome sizes and the presence and absence of several TE families indicated recent lineage invasions. Gene differences between *P. teres* forms are mainly associated with gene-sparse regions near or within TE-rich regions, with many genes possessing characteristics of fungal effectors. Instances of gene interruption by transposons resulting in pseudogenization were detected in PTT. In addition, both forms have a large complement of secondary metabolite gene clusters indicating significant capacity to produce an array of different molecules. This study provides genomic resources for functional genetics to help dissect factors underlying the host–pathogen interactions.

## Introduction

**FIGURE 1:**
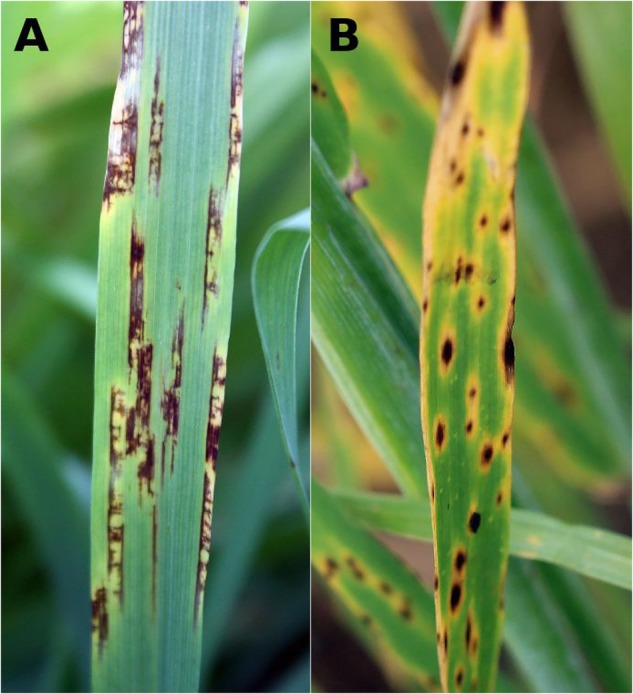
Net blotch field symptoms on barley leaves. **(A)** Net form net blotch (NFNB) caused by *Pyrenophora teres* f. *teres* with lesions along leaf veins containing longitudinal and transverse striations and **(B)** ovoid lesions in spot form net blotch (SFNB) caused by *P. teres* f. *maculata*.

*Pyrenophora teres* [anamorph *Drechslera teres* (Sacc.) Shoemaker] is a haploid ascomycete that causes net blotch in barley. *P. teres* occurs in two forms, *P. teres* f. *teres* (PTT) and *P. teres* f. *maculata* (PTM). PTT disease is known as net form net blotch (NFNB) and is characterized by necrotic lesions along leaves with net-like longitudinal and transverse striations. PTM infection results in spot form net blotch (SFNB), which typically shows discrete ovoid lesions (**Figure 1**). In susceptible cultivars, both diseases spread to encompass entire leaves leading to withering and canopy loss.

While the disease symptoms are different, PTT and PTM are morphologically very similar. A study of intergenic regions suggested a period of evolutionary separation (Ellwood et al., 2012) and their current widespread co-existence in agriculture is thought to be recent. PTT was prevalent before 1970, with Smedegård-Petersen (1971) the first to describe PTM in Denmark and Tekauz reporting PTM in Manitoba, Canada in 1973 (Tekauz and Mills, 1974). PTM appears to have spread since, being noted in Australia in 1977 (Khan and Tekauz, 1982) and in Montana, United States in 1981 (Bockelman et al., 1983). Although artificial hybrids can be readily made in the laboratory (McDonald, 1967; Smedegård-Petersen, 1977a; Campbell and Crous, 2003; Jalli, 2011), PTT and PTM are considered to be genetically autonomous populations (Rau et al., 2003, 2007; Bakonyi and Justesen, 2007; Bogacki et al., 2010; Lehmensiek et al., 2010; Leišová-Svobodová et al., 2014; Akhavan et al., 2016). Natural hybrids appear to be rare and have been suggested by RAPD and AFLP studies (Campbell et al., 2002; Leišova et al., 2005; McLean et al., 2014), and recently confirmed by Poudel et al. (2017) using form-specific markers. However, a lack of field hybrids suggests a natural barrier to fertility as found by Smedegård-Petersen (1977a) or a fitness penalty. The significance of such field hybrids is they may potentially increase population genetic diversity, resistance to fungicides and form new virulence combinations (Campbell et al., 1999; Jalli, 2011) although their contribution in these respects is unknown.

The first genome assembly of PTT was based on 75 bp paired-end Illumina reads (Ellwood et al., 2010). While complex coding regions were effectively assembled, the assembly contained a large number of short scaffolds and contigs of less than 200 bp (*n* > 140,000) and a total assembly size of 41.95 Mbp. Short read assemblies on their own cannot resolve highly repetitive DNA stretches or closely related or identical paralogs (Alkan et al., 2011) and are therefore both shorter and have lower gene counts than reference assemblies. Short read assemblies are also more likely to include instances where single genes are interrupted by low read coverage, leading to mis-assembly and subsequent mis-annotation. Affordable, longer read sequencing technologies are now available that make reference assemblies feasible for biologically important organisms. In the case of PTT and PTM, provision of multiple high quality assemblies will improve dissection of their complex host–pathogen genetic interactions. In addition, such assemblies provide essential information for population diversity studies and a platform for genomics-based gene isolation strategies. Complete assemblies will also broaden our understanding of fungal genomics and contribute to the discovery of common genetic bases to pathogenicity by plant pathogenic fungi.

The objectives of this study were to obtain and compare high-quality reference genome assemblies of PTT and PTM based on single molecule long read DNA sequence data, together with optical and genetic mapping, and to annotate the genomes based on total RNA sequencing data using current best practice annotation practices. The genome assemblies were then used to: explore genomic structural variations, co-linearity and orthology between the *P. teres* forms and between *P. teres* and the related wheat pathogen *Pyrenophora tritici-repentis* (PTR); determine the factor(s) underlying differences in genome size between PTT and PTM; determine the chromosomal locations of repetitive sequences, particularly the association of transposable elements (TEs) with gene coding regions; and finally examine gene content differences between the *P. teres* forms.

The results indicated that both forms of *P. teres* have similar complements of TE families although form-specific differences occur. TEs show more recent activity in PTT leading to a relatively larger genome size than PTM. This is discussed this in the context of the complexity of PTT and PTM host–pathogen interactions and those of similar cereal pathogens. The contribution of TEs to *P. teres* genome architecture and mechanisms underlying genic differences between the forms are also discussed.

## Materials and Methods

### Fungal Isolates

The PTT isolates used in this study were W1-1 collected from the Wongan Hills, Western Australia (WA) in 2009, Stir 9-2 (South Stirlings, WA, 2009), NB29 (Wongan Hills, WA, 1985), NB73 (Tansey, Queensland, 1994), NB85 (Gatton, Queensland, 1995) and a Canadian isolate, 0-1, collected before 1998 (Weiland et al., 1999). PTM isolates used were SG1 (Badgingarra, WA, 1996), Cad 6-4 (Cadoux, WA, 2009), M2 (Muresk, WA, 2009), and FG0B10Ptm1 (FGO) from the United States (Fargo, North Dakota, 2010).

### DNA/RNA Preparation and Sequencing

Single molecule PacBio (Pacific Biosciences, Menlo Park, CA, United States) sequencing requires high molecular weight DNA free of contaminants, a challenge with many fungal species and with *P. teres* in particular as DNA preparations are typically viscous and discolored. Although DNA yields and A260/280 ratios tend to be acceptable with published protocols, A260/230 ratios are consistently low, indicating the presence of polysaccharides. We therefore, incorporated a partial digestion step of fungal cell walls with hydrolytic enzymes. Together with harvesting actively growing tissue, reducing the amount of sugar in growth media, and the use of a high salt extraction buffer, consistently good A260/230 ratios of around two were achieved.

*Pyrenophora teres* starter cultures were grown by scraping surface mycelia from a 1–2 cm^2^ section of a V8 (V8 vegetable juice, Campbells, VIC, Australia) potato dextrose agar plate (V8-PDA). Mycelia were ground with a mini-pestle in a 1.5 ml Eppendorf together with a small volume of sterile water and 100 μl inoculated into 60 ml Fries 2 with 3 g/L sucrose (Fries, 1938). The isolates were grown in the dark at 125 rpm at RT for 2–3 days or until there was adequate biomass. Mycelia were rinsed in sterile water over dairy milk filters (WR&D Wells, Port Melbourne, VIC, Australia), added to 125 ml Fries 2 with 3 g/L sucrose, briefly blended, then grown overnight-24 h as above. Mycelia were harvested by centrifuging at 2000 *g* for 10 min and rinsed three times with mycelial wash solution (1 M NaCl, 10 mM MgCl_2_, 10 mM KH_2_PO_4_ pH 5.8). The number of starter and secondary cultures were scaled according to the growth rates of individual isolates. Two grams of mycelia were digested for 2 h in 40 ml MWS with 3% Extralyse (Laffort, Bordeaux, France) with gentle rocking at 4°C. Mycelia were harvested and rinsed three times with water before drying with milk filters and blotting paper.

DNA was extracted using a modified high salt cetyltrimethylammonium bromide (CTAB) procedure based on that described by Michiels et al. (2003) with the following changes: 0.4 g tissue was used per 7 ml CTAB extraction buffer containing 2 M NaCl. Only a single phenol/chloroform/isoamyl (25:24:1) extraction followed by overnight precipitation with 5 M NH_4_Ac and isopropanol was performed after the CTAB incubation step. To minimize further DNA loss and shearing, a single chloroform/isoamyl (24:1) extraction followed by a 5 M NH_4_Ac and ethanol precipitation was carried out after RNase treatment.

PacBio (Pacific Biosciences, Menlo Park, CA, United States) single molecule, real-time (SMRT) genome sequencing was performed on isolates W1-1, Stir 9-2, NB29, NB73, NB85, and PTM SG1-1 at the McGill University and Génome Québec Innovation Centre (Montreal, QC, Canada). Large insert libraries (∼20 kb) were prepared and sequenced using P6-C4 chemistry in accordance with PacBio protocols.

RNA for isolates PTT W1-1 and PTM SG1-1 were prepared from samples grown *in vitro* in Fries 2 liquid medium and on V8-PDA plates with tissue from germinating spores, exponential growth and sporulation (for V8-PDA plates only). RNA extractions were performed using Trizol (Ambion Life Technologies, Carlsbad, CA, United States) in accordance with the manufacturer’s standard protocol. Any contaminating DNA was removed by DNase I treatment (Ambion) and cDNA synthesized using a BioRad iScript cDNA kit (Bio-Rad, Hercules, CA, United States). One hundred bp paired-end cDNA sequencing was carried out by Macrogen, Inc. (Seoul, ROK) on an Illumina HiSeq 2000. The same sequencing chemistry was used for DNA short read data for isolates W1-1, Stir 9-2, Cad 6-4, M2, and SG1. Summary statistics for DNA and RNA read lengths used in this study are provided in **Table 1**.

**Table 1:** PacBio single molecule long DNA sequencing read and Illumina short read data for *P. teres* isolates used in this study.

	Origin isolate	*P. teres* f. *maculata*				*P. teres* f. *teres*					
		Western Australia			United States	Western Australia			Eastern Australia		Canada
		SG1	Cad 6-4	M2	FGO	W1-1	Stir 9-2	NB29	NB73	NB85	0-1^∗^
PacBio											
	Total read length (Gbp)	6.94	–	–	9.68	5.69	–	9.30	8.82	8.83	9.25
	Estimated DNA read coverage	150	–	–	240	110	–	160	160	160	200
Illumina											
	Total DNA read length (Gbp)	3.61	9.52	3.80	–	6.56	3.84	–	–	–	1.65
	Total RNA read length (Gbp)	9.27	–	–	–	11.49	–	–	–	–	–
	Estimated DNA read coverage	80	120	80	–	120	70	–	–	–	30

*Isolates are grouped by geographic region. ^∗^Indicates based on Wyatt et al. (2017).*

### Optical Mapping

Megabase size DNA from PTT isolate W1-1 was prepared for optical mapping by adapting a protocol for *P. teres* protoplasts (Liu and Friesen, 2012) in combination with a standard method for contour-clamped electric field (CHEF) plugs provided by Opgen (Gaithersburg, MD, United States).

Mycelia were scraped from the surface of a V8-PDA plate and homogenized in a 1.5 ml Eppendorf with a mini pestle. One hundred μl of macerated hyphae were inoculated into 3 ml × 80 ml of Fries 2 liquid medium and grown at RT for 2 days at 125 rpm. Mycelia were harvested by centrifugation at 2000 rpm for 1 min and resuspended in 400 ml Fries 2 with 1/10th (3%) sucrose. The tissue was blended for 30 s with a sterilized immersion blender and grown overnight at RT. The mycelia were harvested by spinning at 2000 rpm for 10 min and washed three times with 100 ml MWS (1 M NaCl, 10 mM MgSO_4_, and 10 mM KPO_4_ pH 5.8). 15–20 g of mycelia were resuspended in 400 ml MWS with 3% Extralyse (Laffort, Bordeaux, France), and incubated overnight at 4°C with gentle rocking.

Protoplasts were filtered through Miracloth (Merck Millipore, Billerica, MA, United States) into a 50 ml Falcon tube. The residual mycelia were rinsed twice with cold MWS while agitating the mycelia with a sterile spatula. This step dislodges protoplasts trapped within mycelia. Protoplasts were centrifuged at 2000 rpm for 10 min and the pellet washed with 40 ml cold STC buffer (1 M sorbitol, 50 mM Tris pH 8, 50 mM CaCl_2_). The protoplasts were then centrifuged at 2000 rpm for 10 min and gently resuspended at concentration of 5 × 10^7^ ml^-1^ to 1 × 10^8^ ml^-1^ in GMB [0.125 M EDTA pH 8, 0.9 M sorbitol (Brody and Carbon, 1989)], followed by incubation at 42°C for 3 min.

To prepare agarose plugs, the protoplasts were combined with an equal volume of 1.5% SeaPlaque agarose (Lonza, Inc., Rockland, ME, United States) in GMB buffer tempered to 42°C and gently mixed by swirling. The suspension was then injected into CHEF plug molds (BioRad, Hercules, CA, United States) and chilled for least 15 min at 4°C. The plugs were incubated in Proteinase K solution (10 mM Tris pH 8, 100 mM EDTA pH 8, 1% *N*-lauryl sarcosine, 0.2% Na deoxycholate, and 1 mg/ml proteinase K) overnight at 50°C, incubated four times for 1 h with 5 ml wash buffer (20 mM Tris pH 8, 50 mM EDTA pH 8), then stored at 4°C in 0.5 M EDTA.

*De novo* whole genome optical mapping of PTT isolate W1-1 was performed by OpGen. The restriction enzyme (RE) *Kpn*I was selected as the most suitable to create a whole genome restriction map based on the average fragment size and composition. The total of 118,123 molecules with a size ≥250KB were used for *de novo* analysis. Proprietary Argus MapSolver^TM^ software v. 3.2.0 was used to build the optical contigs.

### PTT NB29 × NB85 Genetic Mapping

#### Bi-parental Mapping Population

Plates for crossing isolates were prepared by placing autoclaved barley straw pieces in Petri dishes containing Sach’s agar (Hebert, 1971). Plates were inoculated by placing 25 mm^2^ blocks of mycelium from each of two isolates (NB29 and NB85) on opposite sides of the barley straw. Petri dishes were placed into plastic bags to prevent desiccation of the agar and incubated at 15°C with a 12 h light and dark photoperiod. Plates were checked each week for the formation of mature pseudothecia. Once mature pseudothecia were observed, i.e., pseudothecia with a short cylindrical beak, water agar plates were placed on top of the crossing plate with the agar facing the pseudothecia. Plates were sealed with PARAFILM M^®^ (Merck Pty Ltd., Australia) and returned to the incubator. Plates were checked each day for ascospores. Ascospores ejected onto the water agar were removed with a sealed glass needle and single ascospores transferred onto PDA plates. These were incubated at 22°C to allow fungal mycelium to proliferate and DNA extracted using a Wizard^®^ Genomic DNA Purification kit (Promega Corporation, Australia).

#### DArTseq Analysis and Genetic Map Construction

DNA of 89 PTT NB29 × NB85 progeny and the parents was sent to Diversity Arrays Technology (DArT) Pty Ltd. (Canberra, ACT, Australia) for DArTseq^TM^ analysis^1^. DNA samples were processed in digestion/ligation reactions as described in Kilian et al. (2012) with replacement of a single RE *Pst*I-compatible adaptor with two different adaptors with two different RE overhangs. These were a *Pst*I-compatible adapter that incorporated an Illumina flowcell attachment sequence, a sequencing primer site and “staggered,” varying length barcode regions, similar to the sequence reported by Elshire et al. (2011). Reverse adapters contained the flowcell attachment region and *Mse*I-compatible overhang sequence. Only fragments with *Pst*I-*Hpa*II adapters were effectively amplified in 30 rounds of PCR using the following reaction conditions: 94°C for 1 min, 30 cycles of 94°C for 20 s, 58°C for 30 s and 72°C for 45 s and a final step of 72°C for 7 min. After amplification equimolar amounts of the products from each sample were combined and bridge PCR performed with Illumina’s cBot clonal amplification followed by sequencing on an Illumina HiSeq 2000 platform. The sequencing (single read) was run for 77 cycles. Sequences generated from each lane were processed using proprietary DArT Pty Ltd. analytical pipelines. Approximately, 2,000,000 (±7%) sequences per barcode or sample were used in marker calling with DArT Pty Ltd’s proprietary SNP and SilicoDArT (presence/absence of restriction fragments in representation) calling algorithms (DArTsoft14).

MapManager QTXb20 (Manly et al., 2001) was used to partition DArTseq^TM^ markers into linkage groups. RECORD (Van Os et al., 2005) was used to order markers within linkage groups. The Kosambi function was used to calculate map distances. In total 841 markers were assigned to 15 different linkage groups with total genetic map size of 1764 centiMorgans (cM). The average distance between markers was 2.1 cM.

### PTM FGO × SG1 Genetic Mapping

A genetic map of isolate FGOB10Ptm-1 was developed based on a restriction site-associated genotype-by-sequencing (RAD-GBS) approach (Carlsen et al., 2017). The resulting genetic map consisted of 488 filtered SNP markers, with 16 linkage groups and a total map size of 1807 cM. The average distance between markers was 3.7 cM.

### Genome Assemblies

Raw PacBio reads from strains PTT W1-1, PTT NB29, PTT NB85, PTT 73, and PTM SG1 were self-corrected, trimmed and assembled with Canu v1.3 (Koren et al., 2017) using an estimated error rate of 0.03. Where available, Illumina reads were mapped to the Canu assembly using BWA v0.7.15-r1140 (Li and Durbin, 2009). The uniquely mapped reads were filtered using Samtools v1.3.1 (Li et al., 2009) and passed to Pilon v1.17 (Walker et al., 2014) to correct remaining SNP and small indel errors.

The PTT W1-1 reference assembly was scaffolded into chromosomes primarily by matching optical restriction maps against the PacBio contigs. In most cases the optical map provided complete (telomere to telomere) chromosomes, with the genetic map providing a second line of evidence for contig placements and unambiguous orientation of a contig relative to adjacent contigs. A single scaffold join was based on genetic marker evidence to join scaffolds scf01 and scf15. The PTM SG1 reference assembly was enabled by contigs which were collinear with those of PTT with the order supported by genetic map data. Scaffolds were manually merged using Geneious 8.1.8 (Kearse et al., 2012). PTM FGO was assembled as described by Wyatt et al. (2017) for PTT 0-1.

#### Repeat Identification and Masking

Repeats from the seven high quality long-read assemblies were identified using the MAKER recommended method (Campbell et al., 2014). To ensure comparability of results between assemblies and reproducibility of results more generally, the method was formalized as a reproducible Nextflow pipeline (Di Tommaso et al., 2017). The pipeline is available at http://doi.org/10.5281/zenodo.1098651.

Long terminal repeat (LTR) elements were detected using two parallel runs of the LTR harvest (Ellinghaus et al., 2008) and LTRdigest (Steinbiss et al., 2009) packages from Genometools v1.5.8 (Gremme et al., 2013). Recent insertions of LTR elements were detected by setting the similarity cutoff in LTR harvest to 99% and only including repeats with a TGCA palindromic motif sequence. The LTR harvest vicinity cutoff was set to 10 bp to limit the distance searched for target site duplication (TSD). Older LTR instances that are likely to be degraded by genome defense mechanisms such as repeat induced point mutation (RIP) were detected by lowering the LTR harvest similarity cutoff to 85% (the default parameter setting) and removing the requirement that the algorithm must detect an intact copy of the palindromic repeat. In both invocations of LTR digest, the limits of allowable LTR length was 100–6000 bp, the limits of LTR size range was 1.5–25 kbp, and the allowable TSD length was 5 bp. The LTR digest output was passed through the CRL_Step1.pl script distributed with MAKER to remove predictions without both a primer binding site and poly purine tract located in the LTR internal region and to set a maximum distance between LTR and poly purine tract or primer binding site of 20 bp.

False-positives such as local tandem repeats or local gene clusters derived from gene duplications can be detected by a continuation of alignability past the end of the terminal repeats in the LTR instance. To identify and remove these false positives, the CRL_Step1.pl script from MAKER was used to keep only those putative LTR instances without alignability outside of their terminal repeats. LTR instances with nested insertions were also removed and the resulting set used to provide a preliminary masked genome. After LTR elements were removed, RepeatModeler^2^ was run to identify new repeat classes including DNA transposons and LINE elements. Models unable to be identified by RepeatModeler are searched against a database of known transposase sequences. Canonical copies of each repeat class are collected and used as library input the program RepeatMasker. The resulting softmasked contigs were used as input for the genome alignment and annotation.

#### Gene Calling

RNA-seq reads for the reference strains PTT W1-1 and PTM SG1 were treated identically. Reads were trimmed using Cutadapt v1.8.1 (Martin, 2011) to remove adapter sequences and trim read ends below Q30. Trimmed reads were mapped to the corresponding genome assembly using HISAT2 v2.0.4 (Kim et al., 2015) specifying a max intron length of 5 kb and the “dta-cufflinks” option to report alignments tailored for cufflinks input. Cufflinks-guided annotations were generated using CodingQuarry pathogen-mode (CQPM, Testa et al., 2015) which were later provided to Augustus (Stanke et al., 2006; König et al., 2016) as hints. Intron position hints were extracted from the mapped RNAseq reads using the bam2hints utility provided with Augustus and position hints from PTR Pt-1C-BFP proteins (assembly ASM14998v1) mapped to each genome using Exonerate v2.2.0 (Slater and Birney, 2005). Non-coding RNA genes were identified using CMsearch (Cui et al., 2016) using the non-coding RNAs distributed with the companion annotation workflow (Steinbiss et al., 2016).

A rough distance matrix was constructed to inform the whole genome alignment. MinHash distance estimation between each unmasked genome was calculated using MASH (Ondov et al., 2016) and distances converted to a tree using the R-language package phangorn (Schliep, 2010). Softmasked genomes were aligned using progressiveCactus (Paten et al., 2011; Nguyen et al., 2014) to generate a hierarchical alignment (HAL) file. Genes were predicted from the multi-genome HAL dataset using Augustus-CGP (comparative mode) provided with hints describing the position of repeats, CQPM predictions, and PTR Pt-1C-BFP protein hits. Augustus in non-comparative mode was also run over each genome individually using the same hints to generate annotations in regions missed by Augustus-CGP. The final annotations were the union of Augustus and Augustus-CGP runs, giving preference to the Augustus-CGP output in the case of conflict as calculated with BEDtools v2.25.0 (Quinlan and Hall, 2010). Orthologous protein sets between *P. teres* isolates in this study and PTR strain Pt-1C-BFP (Manning et al., 2013) were detected using Orthofinder v1.1.8 (Emms and Kelly, 2015).

Whole genome alignments, annotations, RNA seq, and other data sources were visualized as a custom track hub on the UCSC genome browser (Tyner et al., 2016) which was used to manually check annotation results. The UCSC genome browser and Mummer (Kurtz et al., 2004) were used to visualize syntenic relationships between assemblies.

### Detection of *P. teres* Biosynthetic Gene Clusters

In order to investigate the presence and diversity of the *P. teres* biosynthetic gene clusters (BGCs) involved in specialized metabolism (SM), the PTT W1-1 and PTM SG1 reference genomes were processed through the web based fungal version of the Antibiotics and Secondary Metabolite Analysis Shell (AntiSMASH) v. 4.0.0rc1 (Medema et al., 2011; Blin et al., 2017) as unannotated fasta files. ‘Basic’ and ‘deep’ analyses were performed. The ‘basic’ analysis used only KnownClusterBlast, SubClusterBlast, smCoG analysis, ActiveSiteFinder, and whole-genome PFAM analysis in addition to the core modules. In addition, the ‘deep’ analysis was performed by activating the ClusterFinder module along with its algorithm for BGC border prediction, with the additional features of ClusterBlast and Cluster-border prediction based on transcription factor binding sites. The parameters for ClusterFinder were a minimum cluster size of five CDS, five biosynthesis-related PFAM domains, and a minimum ClusterFinder probability score for a BGC of 80%.

### Data Availability

DNA read files, reference assemblies and metadata for Australian *P. teres* isolates in this study are available under European Bioinformatics Institute accession PRJEB18107. The PTT isolate 0-1 genome assembly was submitted to NCBI GenBank under BioProject PRJNA392275 and PTM FGO under PRJNA417860.

## Results

Representative isolates of PTT (isolate W1-1) and PTM (SG1) from Western Australia were used to develop high quality reference assemblies. For downstream analyses and to provide unambiguous evidence for *P. teres*-form specific genomic features and for comparative genomics, an additional five PacBio assemblies from geographically distinct regions (the eastern states of Australia and North America) were included. These isolates were originally selected for sequencing as they represent parents in genetic mapping populations. Three short-read Illumina assemblies, including two PTM isolates for which just two PacBio assemblies were established, were also included to validate gene presence/absence between forms and to avoid erroneous gene calls caused by isolate-specific SNPs.

Gene call accuracy was improved by *in silico* reversal of RIP-like mutations before repeat detection and masking. Older repeat instances subjected to RIP G/C-to-A/T transition mutations diverge away from the original canonical form. The introduction of enough sequence variation can therefore frustrate detection and classification of degraded repeat instances. Without detection and masking of these elements, spurious gene annotations can inflate gene count and inter-form gene differences.

### Resolution of Full-Length *P. teres* Chromosomes

The combined long DNA PacBio, genetic and optical maps resulted in high quality genome assemblies of 41.58 and 51.76 Mbp for the nuclear genomes of PTM SG1 and PTT W1-1 (**Table 2**). Size ranges based on two PTM genome assemblies and five PTT assemblies were consistently different between the forms. PTM is smaller, ranging from 39.27 to 41.28 Mbp, with PTT ranging from 46.31 to 51.76 Mbp. Estimated PTM genome sizes from two additional short-read only assemblies were also consistently smaller, indicating the total genome size differences are not specific to the two PTM isolates chosen for PacBio genome assemblies. Both *P. teres* forms comprise 12 chromosomes with no evidence of chromosomal fusion or rearrangements between them. Consistent with total genome sizes, the chromosomes ranged in size from 1.99 to 7.22 Mbp in PTT W1-1 and 1.56–5.43 Mbp for PTM SG1. Chromosomes were numbered to match the PTT 0-1 chromosomes (Wyatt et al., 2017).

**Table 2:** Summary genome assembly characteristics for isolates sequenced by PacBio.

Isolate	*P. teres* f. *maculata*		*P. teres* f. *teres*				
	SG1	FGO	W1-1	0-1^∗^	NB29	NB73	NB85
Chromosomal Scaffolds^∼^	12 (97.09)	13 (97.20)	12 (96.37)	12 (92.21)	–	–	–
Contigs^∧^	47 (21)	46 (17)	74 (45)	85 (42)	55	43	47
Assembly length (Mbp)	41.28	39.27	51.76^#^	46.31	50.12	48.03	49.03
N_50_ (Mbp)	2.11	1.46	3.70	1.73	3.27	3.17	3.32
L_50_	7	9	6	11	6	6	6
GC content (%)	46.86	47.37	45.21	46.68	45.57	45.94	45.71
Length GC-equilibrated regions (Mbp)	32.73	32.62	36.34	34.79	36.07	35.08	35.58
Genes	11,165	11,080^+^	11,245	11,173^+^	11,214	11,199	11,193

*Reference nuclear genomes based on supporting genetic mapping data, optical mapping data (W1-1 only), and RNA-guided gene calls are shaded gray. ^∼^Whole chromosome scaffold number is given with the percentage of the whole genome in brackets. ^#^Estimated nuclear genome size based on optical map data is 53.08 Mbp. ^∧^The number of unplaced contigs for scaffolded assemblies is given in brackets. ^∗^Based on Wyatt et al. (2017), or this study^+^. L_50_ = the smallest number of contigs that contain at least 50% of the assembly length, N_50_ = length of the smallest contig in the L_50_ set.*

All the assemblies contained unplaced contigs, for example 45 for PTT W1-1 and 21 for PTM SG1, representing 4% or less of the assemblies. In PTT W1-1, 20 of these were below the cutoff size for the optical mapping service provider (40 kb). All unplaced contigs were characterized by low G/C regions or transposon-like repeats. For PTT W1-1, a total of 24 telomeres were detected in either the assembly or optical map, corroborating 12 complete chromosomes. Nineteen of these are illustrated in Supplementary Figure 1, with the remainder detected on unplaced contigs. In PTM SG1, in the absence of optical mapping, only 10 telomeres were detected on the whole chromosome scaffolds with four present in the unplaced contigs. However, an identical chromosome complement is evident as the whole chromosome scaffolds are congruent with PTT W1-1. As each PTT W1-1 chromosome has a corresponding chromosome in PTM SG1, no dispensable or accessory chromosomes are inferred.

### Macrosyntenic Relationships and Genome Fissuring Between PTT, PTM, and PTR

**FIGURE 2:**
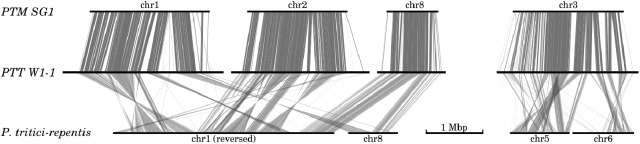
Large scale inter- and intra-chromosomal rearrangements between *P. teres* and *P. tritici-repentis*. Two major rearrangements were detected between both forms of *P. teres* and *P. tritici-repentis*. Nucmer matches (Kurtz et al., 2004) were filtered to show regions longer than 1 kb.

Genome-wide comparison with the PTR strain Pt-1C-BFP assembly (Manning et al., 2013) revealed widespread megabase-scale intra-chromosomal inversions when compared against both *P. teres* forms (**Figure 2**). Translocation breakpoints underlying the inversions were difficult to resolve due to the frequency of scaffold gaps in the PTR assembly. For example 157 gaps are present in PTR chromosome 1, and 45 in chromosome 8. Of the 31 resolvable translocation breakpoints between PTR and PTT W1-1, 27 occur within 10 kb of annotated LTR repeat instances. Significantly, two large scale *inter*-chromosomal events were observed between PTR and both *P. teres* forms (**Figure 2**). Inverted and collinear rearrangements of large syntenic blocks between chromosomes 1, 2, and 8 from both PTT and PTM are evident with PTR chromosomes 1 and 8. The second major inter-chromosomal event involves chromosome 3 in *P. teres*, which is composed of approximately half of PTR chromosome 5 and half of PTR chromosome 6 (**Figure 2** and Supplementary Figure 2).

**FIGURE 3:**
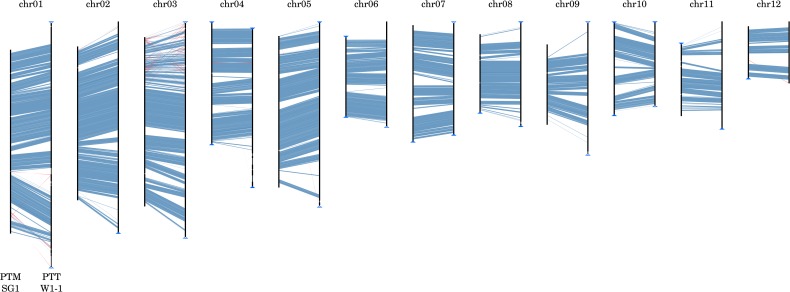
Whole genome macrosyntenic comparison of *P. teres* f. *teres* and *P. teres* f. *maculata*. Nucmer matches (Kurtz et al., 2004) show high conservation of synteny between the two forms, represented here by strains PTM SG1 (chromosomes depicted on the left-hand side of each alignment) and PTT W1-1 (chromosomes depicted on the right-hand side). Collinear syntenic blocks >1 kb in length are shown in blue. Gaps between syntenic blocks are composed of low GC, repetitive, and transposon-rich regions. Similarly, matches to the reverse strand shown in red are repetitive transposon-rich regions rather than genic translocations or inversions.

The *P. teres* forms, like many of the Pezizomycotina, exhibit features characteristic of “two-speed” genomes (Dong et al., 2015), with stretches of GC-equilibrated, gene-rich regions separated by repetitive, AT-rich isochores. The GC-equilibrated regions are highly syntenic (**Figure 3**) whereas the AT-rich isochores are highly variable in both length and presence (as exemplified in Supplementary Figure 5). Expanded and unique AT-rich isochores form distinctive wedge-shaped alignments when comparing isolates and are referred to herein as genomic ‘fissures.’ In general, the number of such fissures and their length are both higher in PTT relative to PTM and account for both within-form and between-form genome size differences. In PTT W1-1, more recent TE activity is indicated by AT-rich isochores with a length greater than 10 kbp being more frequent than in PTM SG1 (546 vs. 272) but shorter on average (mean lengths 91 and 150 kbp, respectively).

### Repeat Contents and the Tad-1 LINE-Like Element Abundance Differentiate the Two Forms

**FIGURE 4:**
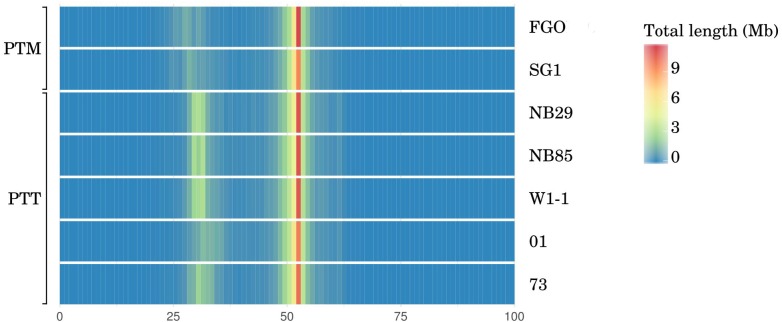
Whole-genome histogram of GC-content for all *P. teres* PacBio genome assemblies generated by OcculterCut (Testa et al., 2016). In both *P. teres* f. *teres* (PTT) and *P. teres* f. *maculata* (PTM), in all genomes, the bulk of the genome is GC-equilibrated to just over 50%. Secondary bands with approximately 30% GC content are stronger in PTT than PTM. GC content of DNA sequencing reads are shown in Supplementary Figure 4. The intensity of secondary bands vary between PTT isolates consistent with genome size.

The chromosomal-scale differences between *P. teres* forms is almost entirely due to expansion in repetitive elements. The sum length of repetitive DNA between PTT isolates, inferred from the non-GC equilibrated fraction, varies by 4 Mbp. Moreover, the mean length of the long-read PTT genome assemblies is 21% longer than PTM and the length of GC-equilibrated sequence representing coding DNA and recent TE insertions, estimated by OcculterCut (Testa et al., 2016), is 9% longer in PTT than PTM (**Table 2**, with whole genome GC distribution graphically shown in **Figure 4**).

**FIGURE 5:**
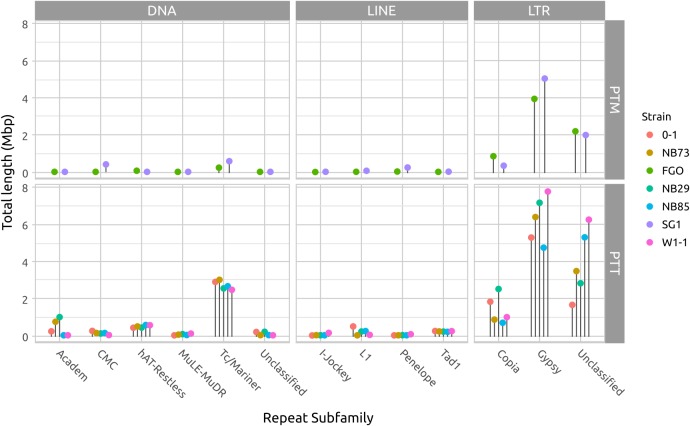
Total repeat length of each repeat element type. The difference in genome size between *P. teres* f. *teres* (PTT) and *P. teres* f. *maculata* (PTM) is due to a relatively larger length of low-GC regions in PTT. In both forms, long terminal repeats (LTRs) comprise the bulk of the repeats. The DNA transposable Tcl-Mariner-like elements are consistently more abundant in PTT than PTM. A table of repeat lengths for two isolates is available in Supplementary Table 1 and a log-transformed version or this figure presented in Supplementary Figure 3 to show low abundance families.

Long DNA read genome assemblies enable a more complete catalog of the repetitive DNA present in each isolate than previous short-read assemblies. The largest contributors to repeat composition in both forms are LTR elements, composed principally of Gypsy and Copia families, and DNA transposon Tc1/Mariner elements (**Figure 5** and Supplementary Table 1). Copia and Tc1/Mariner elements in particular are more frequently observed in PTT than PTM. With the exception of Tc1/Mariner TE’s, DNA transposons and LINE-like TE’s occur less frequently than LTR TE’s. The hAT-Restless and Tad-1 families are more abundant in PTT than PTM (Supplementary Figure 3). Tad-1 is present in negligible amounts in PTM (average total length 2 kb), but consistently present in PTT (average total length 214 kb). Very low abundance repeat element classes are likely to be spurious miss-classification of small fragment matches due to extensive RIP degradation of older TE’s.

### Gene Differences Between *P. teres* Forms Are Due to TE Activity and Interruption Resulting in Pseudogenization or Presence/Absence Genotypes in TE-Rich Regions

The number of predicted genes both between and within PTT and PTM are similar (**Table 2**). The vast majority of *P. teres* genes lie within GC-rich syntenic regions and are more stable and conserved than genes influenced by TE-mediated genome fissuring. One hundred and twenty two genes differentiated the two forms, with many (34%) satisfying the criteria for small secreted proteins typical of fungal effector proteins (possessing a signal peptide, less than 50 kDa, and cysteine-rich). One hundred and fourteen of the gene set were located in regions of low gene density proximal to, or as GC-rich islands within, repetitive regions.

Nine genes were functional absences after interruption by TE insertion. TE insertion is both a potential mechanism for the generation of pseudogenes by interruption of coding sequence, and a mechanism to inactivate genes. The increased TE complement and activity in PTT relative to PTM appears to have increased the frequency of gene interruption. Typically, such events resulted in the automated annotation of two likely erroneous genes either side of the insertion which require manual curation. Interrupted genes included a heterokaryon incompatibility protein, a FAD-binding oxidoreductase, a member of the glucose–methanol–choline (GMC) oxidoreductase family, and six genes with uncharacterized products. The size of the interrupting insertions ranged from 169 to 6,514 bp.

### *P. teres* Possesses an Expanded Array of Non-ribosomal Peptide Biosynthetic Gene Clusters

Early research on barley net blotch established that *P. teres* isolates produce small molecules with phytotoxic activity (Smedegård-Petersen, 1977b; Coval et al., 1990). Although none of the described molecules are major disease drivers, they may contribute to pathogenicity since their effects correlate with chlorotic symptoms of net blotch of barley.

The reference genomes of PTM W1-1 and PTT SG1 were therefore examined for the abundance and type of BGCs, responsible for the production of small molecules via specialized metabolism pathways (SM, also referred to as secondary metabolites but indicating molecules with a significant ecological, symbiotic or pathogenic role rather than basic or common cellular function). There are several SM biosynthetic pathways but those producing non-ribosomal peptides (NRPs), polyketides (PKs), PKS-NRP hybrids, and terpenes are the most prevalent. **Table 3** summarizes the SM BGCs found in the reference genomes by AntiSMASH analyses. In a ‘basic’ analysis a total of 66 and 41 gene clusters were found for PTT and PTM, respectively. Interestingly, NRPs are over-represented in PTT compared to PTM and other ascomycete fungal genomes which tend to number around 10 (Amselem et al., 2011). While PTM has 11 NRPs, PTT has more than thrice as many, 38 which represents more than half of all its BGCs. Additionally, 10 PKS-NRPs hybrid BGCs are present in both PTT and PTM reference genomes. When performing the ‘deep’ analysis using the ClusterFinder algorithm, two fatty acid related SM BGC and around 25 extra putative BGCs of uncharacterized SM type were predicted per genome, yielding a total of 97 clusters for PTT and 68 for PTM.

**Table 3:** Number of BGCs found within PTT (W1-1) and PTM (SG1) genomes using AntiSMASH.

Isolate	PKs	NRPs	PK-NRPs	Terpenes	Other^∧^	*Clusterfinder*	Total
PTT (W1-1)	12	38	10	4	2	31	97
PTM (SG1)	11	11	10	5	4	27	68

*The number of BGCs found with the ‘basic’ AntiSMASH (Medema et al., 2011; Blin et al., 2017) analysis are tabulated for each category: Polyketides (PKs), non-ribosomal peptides and non-ribosomal peptide-like (NRPs), PK-NRPs hybrids, terpenes, and other (with ^∧^indicating these BGCs that do not conform to the main classes). The column ‘Clusterfinder’ presents additional BGCs found with the AntiSMASH ‘deep’ analysis. The total number of BGCs predicted per genome from both ‘basic’ and ‘deep’ analyses are presented in the last column.*

## Discussion

### Genome Size Reflects Shared Genomic Lineage and the Degree of Host–Pathogen Specialization in *Pyrenophora* spp.

Several articles have correlated genome size with the lifestyle of plant pathogenic and non-pathogenic fungi (e.g., Raffaele and Kamoun, 2012; Dong et al., 2015). Fungal crop pathogens are hypothesized to have larger genomes than their free-living relatives with rapidly evolving repetitive regions serving to generate pathogenesis effectors. Among filamentous host-specific cereal leaf pathogens, biotrophs possess the largest and most repetitive genomes, with *Blumeria graminis* f. sp. *tritici*, for example, at 159 Mbp^3^.

The PacBio assemblies of PTM and PTT in this study averaged 40 and 49 Mbp, respectively, a clear difference for which an association with pathogen lifestyle might be predicted. Necrotrophs are defined as organisms that kill host cells and feed saprotrophically on the dead matter and hemibiotrophs as organisms with an initial period of biotrophy followed by necrotrophy, though there may be a continuum of transitional states. Both PTM and PTT have relatively large, multicellular conidia with which to initiate infection and both have a short asymptomatic phase of around 48 h before necrotic lesions develop. During this phase, PTM forms haustorial-like intracellular vesicles (Lightfoot and Able, 2010), suggesting biotrophic-like acquisition of nutrients. Such structures were rarely observed in PTT, which grows more extensively on the leaf surface before growing throughout the mesophyll, although they are not a prerequisite of biotrophism (Spanu and Panstruga, 2017). Both *P. teres* forms may therefore be regarded as hemibiotrophs.

Host–pathogen genetic interactions indicate that PTT is more intimately adapted to barley. In both interactions, host resistance is complex with numerous quantitative gene resistances effective at seedling, adult and all stages across the barley genome (McLean et al., 2009; Liu et al., 2011). Pathogen virulence involves different QTL of varying effect, however, stronger differential responses to PTT occur and virulence in PTM is more quantitative and additive (Shjerve et al., 2014; Carlsen et al., 2017; Koladia et al., 2017). Host resistance to PTM also appears to be characterized by quantitative minor gene resistance only. In PTT, stronger differential reactions involve dominant host susceptibility genes, which govern necrotrophic effector–host interactions, and dominant resistance genes, with sudden and dramatic breakdowns in cultivar resistance suggestive of major dominant resistance genes more typical of biotrophic interactions.

Among other *Pyrenophora* species sequenced to date, the wheat necrotroph *P. tritici-repentis* possesses a predicted genome size of approximately 40 Mbp (Manning et al., 2013), recently corroborated by a PacBio assembly at 40.8 Mbp (Caroline Moffat, personal communication). The unspecialized grass weed seed pathogen *P. semeniperda* has an estimated genome size of 40.1 Mbp based on 454 Life Sciences sequencing (Soliai et al., 2014). Both PTR and PTM have a relatively recent association with their crop hosts. PTR was occasionally found on wheat before the 1940s and became a significant pathogen following the horizontal transfer of the effector ToxA from *Stagonospora nodorum* (Nisikado, 1929; Barrus, 1942; Friesen et al., 2006). PTM spread world-wide from the 1970s onwards (McLean et al., 2009) and may have been present on barley in Japan as early in the 1930s (Ito and Kuribayashi, 1931). PTR and PTM share broad epidemiological similarities with *Ramularia collo-cygni*, a barley pathogen that has been known for over 100 years but has only become an important pathogen in the last 30 years (Havis et al., 2015). Like PTR and PTM, *R. collo-cygni* is genetically diverse, indicating sexual reproduction during the growing season, and also undergoes clonal proliferation. Although not as well-studied, quantitative variation in resistance occurs suggesting polygenic minor gene resistance. However, the *R. collo-cygni* genome is only 30 Mbp (McGrann et al., 2016).

Limited periods of co-evolution of PTR and PTM with their crop hosts, the complexity of their genetic interactions and shared ancestry may explain their smaller genomes alongside *P. semeniperda*. Disease is primarily governed by just three effectors in PTR (Ciuffetti et al., 2010) and by iterative minor effect QTL in PTM (Carlsen et al., 2017). In recently invaded crops, a formerly marginal host (one that is not normally susceptible to a pathogen but with occasional susceptible genotypes) may lead to partial suppression of basal resistance (Niks and Marcel, 2009). Such genotypes may serve as a basis for further pathogen adaptation and a platform for colonization in the context of modern agriculture, whereby the widespread use of a limited number of cultivars creates high selection pressure. This may explain the common epidemiologies of PTR, PTM and *R. collo-cygni* and the apparently static PTR and PTM genome sizes.

### Tad-1 and Tc1/Mariner TE’s Distinguish PTT and PTM

Comparing TE presence and absence even after de-ripping is problematic due to nested insertions, the extent of RIP, and spontaneous mutations in older repetitive sequences (Hane and Oliver, 2010). Individual *P. teres* isolates also appear to have slightly different compliments of TE’s so that if only one isolate of each form are compared, a greater number of familial differences may be indicated than between several isolates (Supplementary Figure 3). Subject to this caveat and that only two PTM isolates were included in comparisons, one family, the LINE-like Tad-1, was exclusive to PTT. A low number of Tad-1 like sequences were present in PTM SG1 but were short and predominantly <300 bp, compared to full length elements of 7 kb (Cambareri et al., 1994), indicating inactive vestigial elements or possibly spurious matches. The large variation in genome sizes within PTT, notably between isolates W1-1 and 0-1, is reflected in the abundance range of different TE’s. However, a greater abundance of the DNA transposon Tc1/Mariner in PTT also appears to differentiate the forms (**Figure 3**).

### *P. teres* Genome Architecture Driven by TE-Mediated Translocation

The genomes of PTT and PTM are highly collinear, at both the macrosyntenic (conservation of synteny across large sections of a chromosome) and microsyntenic (individual gene order) levels. The collinearity is interrupted by TE-rich isochores which are usually expanded in PTT relative to PTM. The gene-rich regions are also highly syntenic when compared to PTR (**Figure 2**), suggesting these regions have been shuffled as single units since divergence of PTR and *P. teres*. This confirms the phylogeny found by Ellwood et al. (2012), where an ancestral form of *P. teres* diverged from PTR for a substantial period of time with the two forms diverging only relatively recently. This also indicates an invasion of TE elements in *P. teres* ancestral forms prior to the divergence of PTT and PTM provided the opportunity for shuffling of these islands, followed by expansion in the length of the TE-rich isochores in PTT subsequent to divergence from PTM.

Ohm et al. (2012) suggested that the breakpoints of chromosomal inversion in the Dothideomycetes are associated with SSRs, but more recent evidence suggests that breakpoints are closely associated with LTRs at least in the case of *Leptosphaeria maculans* (Grandaubert et al., 2014) and *Parastagonospora nodorum* (Syme et al., unpublished data). A comparison of breakpoints in PTT and PTM relative to PTR (data not shown) confirmed the LTR association. This study and that by McGrann et al. (2016) also confirm that a phenomenon known as ‘mesosynteny,’ the conservation of gene content but not order in chromosomes across widely divergent fungal genera (Hane et al., 2011), does not occur between *Pyrenophora* species. Whether this is genus-specific or simply a result of older and less complete assemblies failing to accurately identify interchromosomal translocations remains to be resolved.

Chromosomal rearrangements driven by TE expansion can reduce fertility and contribute to reproductive isolation (Delneri et al., 2003; Coghlan et al., 2005). In the case of *P. teres*, the expansion of repetitive elements in PTT has not yet contributed to chromosomal rearrangements between the two forms, so low rates of survival of natural hybrids between the forms in the field (Poudel et al., 2017) cannot be explained by such rearrangements. The viability of PTT/PTM hybrids is therefore more likely due to unequal recombination events leading to deleterious gene loss or gain, or a fitness penalty associated with incompatibility between new gene combinations, or another yet to be determined mechanism rather than large-scale genome dynamics.

### NRP Clusters Are Expanded in PTT

We analyzed the presence of BGCs within the *P. teres* reference genome assemblies as small molecules have been associated with disease among the Dothideomycetes (Stergiopoulos et al., 2013; Muria-Gonzalez et al., 2015) and in *P. teres* contribute to chlorosis (Smedegård-Petersen, 1977b; Coval et al., 1990). Significantly, PTT isolate W1-1 has the largest number of NRPs and PK-NRPs BGCs yet reported. These may have evolutionary significance since PTT’s most phytotoxic NRP alkaloids, *N*-(2-amino-2-carboxythyl)-aspartic acid (toxin A) and aspergillomarasmine A (toxin C), have been associated to disease severity (Bach et al., 1979; Weiergang et al., 2002). Interestingly, many of the predicted NRP BGCs in the genomes of *P. teres* are non-canonical since some of the main enzymatic domains (e.g., adenylation, peptidyl carrier protein, condensation, and/or thioesterase domains) appear to be missing. In the human pathogen, *Aspergillus fumigatus*, a NRP synthase-like enzyme lacking a condensation domain produces isoquinoline alkaloids, the fumisoquins (Baccile et al., 2016). Plausibly, *P. teres* NRP-like BGCs are also responsible for the production of non-NRP alkaloids such as the non-host specific isoquinolines, pyrenolines A and B (Coval et al., 1990).

While a more in depth analysis of the BGC genes of PTT and PTM are required to better evaluate their contribution to pathogenicity, our results show the enormous capacity of these fungi to produce an array of different molecules. Furthermore, the additional uncharacterized SM BGC predicted by the ‘deep’ analysis highlights a limitation of defining BCGs based on better characterized models involved in NRPs and PKs biosynthesis.

### Detection of TE-Mediated Gene Disruption

To provide an evolutionary advantage that outweighs the cost of their maintenance, repeat-rich compartments introduce highly variable and expanded intergenic space that can serve as hotbeds of adaptive evolution (Dong et al., 2015). TEs can provide mobility to effector genes, either within a genome to regions of increased transcriptional activity (Grandaubert et al., 2014), or between genomes to expand host range (Friesen et al., 2006). TEs can influence nearby genes, including pathogenicity elements by introducing variation to coding sequence by incomplete excision or via RIP leakage (Daboussi et al., 1992; Fudal et al., 2009; Rouxel et al., 2011; Daverdin et al., 2012). RIP leakage generates not only new functional effector genotypes but can inactivate a gene by introducing premature stop codons (Daboussi et al., 1992).

When the initial transposon insertion occurs in a genic region, the insertion event may interrupt coding sequence and result in a truncated and likely non-functional gene. Genes interrupted by TE-mediated fissuring may be misidentified in single-genome studies due to mis-annotation. Interrupted and non-functional genes over time also accumulate mutations, a process that may be accelerated by RIP leakage. While there are still relatively few mutations, the interrupted sequence may retain the statistical properties of coding sequence used by most gene annotation algorithms. The detection of a recent gene interruption is therefore difficult without multiple genome comparisons, as fragments produced by a fissuring event will still retain the nucleotide composition of coding sequence and are likely to be annotated as one or two genes either side of the repetitive sequence unless a well-assembled genome with an uninterrupted locus is also available. These potentially erroneous annotations may lead to two types of error in gene-based comparative genomics; the total gene count is inflated in the interrupted strain together with the count of strain-specific genes. Complicating matters further is the possibility that interruption of a gene by TEs to produce a truncated protein may be a source of new effector-like genes, so if a fissuring event is detected, it is not easy to ascertain whether the divided loci are pseudogenes or one or two new small, functional genes.

### Genic Features Particular to Each *P. teres* Form

Genic differences between the forms were found to be associated mainly with TE-rich regions. Several instances of genes interrupted by TE insertions were apparent in the genome assemblies and the remainder are genes that have been inserted or lost from one form or the other. Interrupted genes were exclusively found in repetitive regions of the PTT genome with the intact form present in PTM isolates. The majority of these genes (six out of nine) are of unknown function.

Form-specific genes are also prevalent in regions of low gene density – small islands of less than 10 genes adjacent to AT-rich repeat sections. These isolated genes often share characteristics of fungal effector genes including encoding small proteins, a high cysteine content and the presence of a secretion signal. Secreted effectors are at risk of detection by host resistance genes and therefore effectors that can be easily altered or lost to evade recognition provide genetic agility should the pathogen encounter a host genotype with novel resistance loci. The isolation and proximity to repetitive sequence of the form-specific loci may lead to sequence alteration through RIP leakage as described above or facilitate their loss by looping out during meiosis. These mechanisms may contribute to the presence/absence genotype characteristic of necrotrophic fungal effector genes. In addition, interruption by TEs is also an effective mechanism for removing avirulence genes while also producing new, smaller genes that may provide a basis for generating new effector-like genes.

## Author Contributions

RS performed all genome assemblies and bioinformatic analyses. NW and TF provided the FGOB10Ptm-1 assembly and helpful comments on the manuscript. AM developed the *P. teres* f. *teres* mapping population and conducted genetic mapping. MM-G compared metabolite gene cluster differences. SE and JL developed the high MW DNA protocol. SE wrote the manuscript.

## Conflict of Interest Statement

The authors declare that the research was conducted in the absence of any commercial or financial relationships that could be construed as a potential conflict of interest.

## Footnotes

1 http://www.diversityarrays.com/

2 http://www.repeatmasker.org

3 fungi.ensembl.org/Blumeria_graminis/Info/Index
