# Supplementary material for: Transposable Element Genomic Fissuring in Pyrenophora teres Is Associated With Genome Expansion and Dynamics of Host–Pathogen Genetic Interactions
Source: Front Genet. 2018 Apr 18;9:130. doi: 10.3389/fgene.2018.00130 (PMC5915480; doi:10.3389/fgene.2018.00130)
Supplement: Supplementary file 1 [file Table_1.DOCX]

# Supplementary Material

##

## Supplementary Table 1. Composition and identity of TE families in *P. teres* isolates SG1 and W1-1. Contribution of TE families belonging to Class I (LTR and LINE transposons) and Class II (DNA transposons) to the repetitive genome fraction of isolates PTT W1-1 and PTM SG1.

|  |  | **Total length (bp)** | | **Percentage of total repeat content** | | **Percentage of total assembly length** | |
| --- | --- | --- | --- | --- | --- | --- | --- |
|  |  | SG1 | W1-1 | SG1 | W1-1 | SG1 | W1-1 |
| **LTR Retrotransposon** | | | | | | | |
|  | Copia | 316,055 | 992,051 | 3.6% | 5.4% | 0.8% | 1.9% |
|  | Gypsy | 5,026,516 | 7,735,213 | 57.9% | 41.7% | 12.2% | 14.9% |
|  | Unclassified | 1,981,936 | 6,237,597 | 22.8% | 33.7% | 4.8% | 12.1% |
|  | **Total** | 7,324,507 | 14,964,861 | 84.3% | 80.7% | 17.7% | 28.9% |
| **LINE** | | | | | | | |
|  | I-Jockey | 0 | 140,744 | 0.0% | 0.8% | 0.0% | 0.3% |
|  | L1 | 67,944 | 31,327 | 0.8% | 0.2% | 0.2% | 0.1% |
|  | Penelope | 227,203 | 59,842 | 2.6% | 0.3% | 0.6% | 0.1% |
|  | Tad1 | 2,296 | 221,971 | 0.0% | 1.2% | 0.0% | 0.4% |
|  | **Total** | 297,443 | 453,884 | 3.4% | 2.4% | 0.7% | 0.9% |
| **DNA Transposon** | | | | | | | |
|  | CMC-EnSpm | 404,053 | 11,136 | 4.7% | 0.1% | 1.0% | 0.0% |
|  | hAT-Restless | 24,590 | 545,071 | 0.3% | 2.9% | 0.1% | 1.1% |
|  | MuLE-MuDR | 35,402 | 102410 | 0.4% | 0.6% | 0.1% | 0.2% |
|  | Tc1/Mariner | 571,043 | 2,456,443 | 6.6% | 13.3% | 1.4% | 4.7% |
|  | Unclassified | 26,764 | 0 | 0.3% | 0.0% | 0.1% | 0.0% |
|  | **Total** | 1,061,852 | 3,115,060 | 12.2% | 16.8% | 2.6% | 6.0% |
