# Supplementary material for: Transposable Element Genomic Fissuring in Pyrenophora teres Is Associated With Genome Expansion and Dynamics of Host–Pathogen Genetic Interactions
Source: Front Genet. 2018 Apr 18;9:130. doi: 10.3389/fgene.2018.00130 (PMC5915480; doi:10.3389/fgene.2018.00130)

## Supplementary Figure 1. Chromosomal assemblies for reference strains PTT W1-1 and PTM SG1 showing relative sizes together with the position and size of scaffold gaps in the genome assemblies. Gaps between contigs in PTT W1-1 are estimated from optical map distances. Gap sizes in SG1 are unknown, so are not to scale and were set at 100 bp.


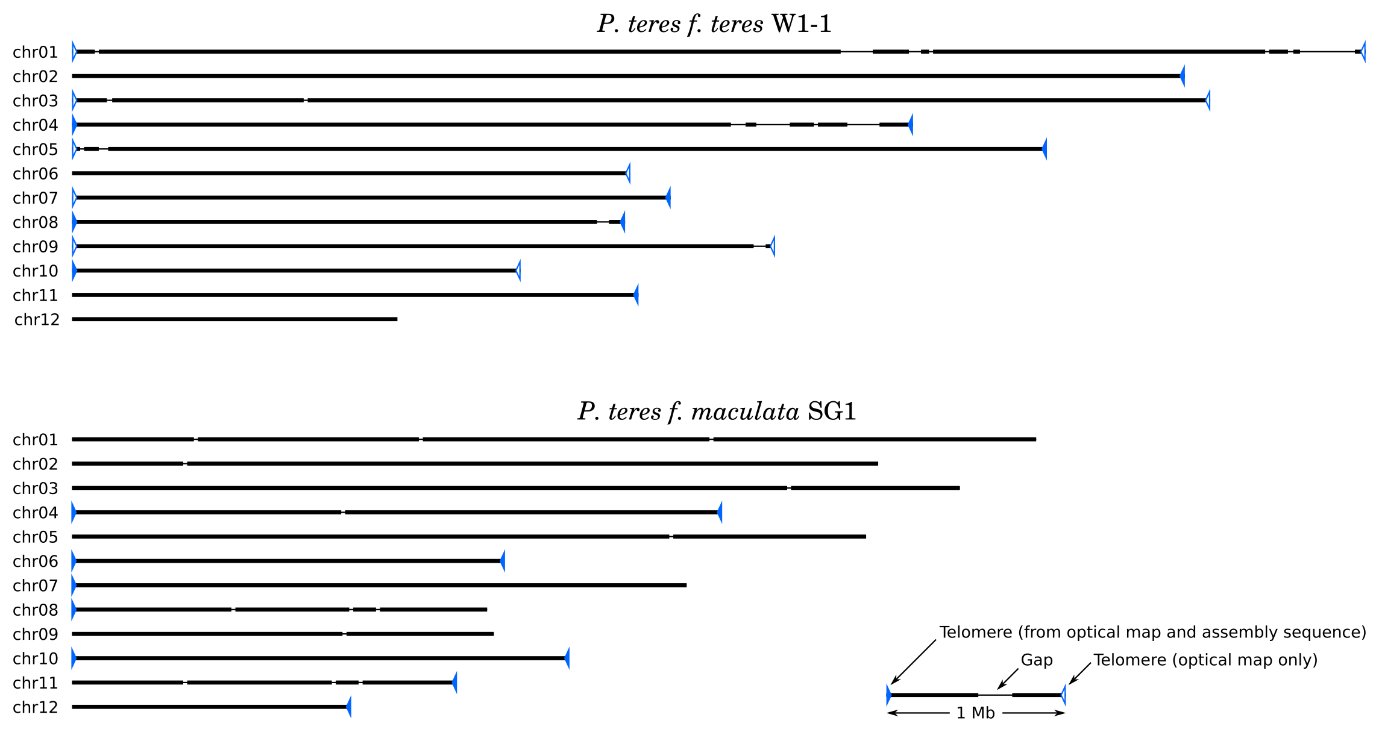

Supplement: Supplementary file 2 [file Data_Sheet_1.DOCX]
