# Supplementary material for: Transposable Element Genomic Fissuring in Pyrenophora teres Is Associated With Genome Expansion and Dynamics of Host–Pathogen Genetic Interactions
Source: Front Genet. 2018 Apr 18;9:130. doi: 10.3389/fgene.2018.00130 (PMC5915480; doi:10.3389/fgene.2018.00130)

## Supplementary Figure 2. Nucmer match dotplot (Kurtz et al. 2004) showing syntenic relationships between *P. tritici-repentis* Pt-1C-BFP and *P. teres* f. *teres* W1-1 chromosomes.


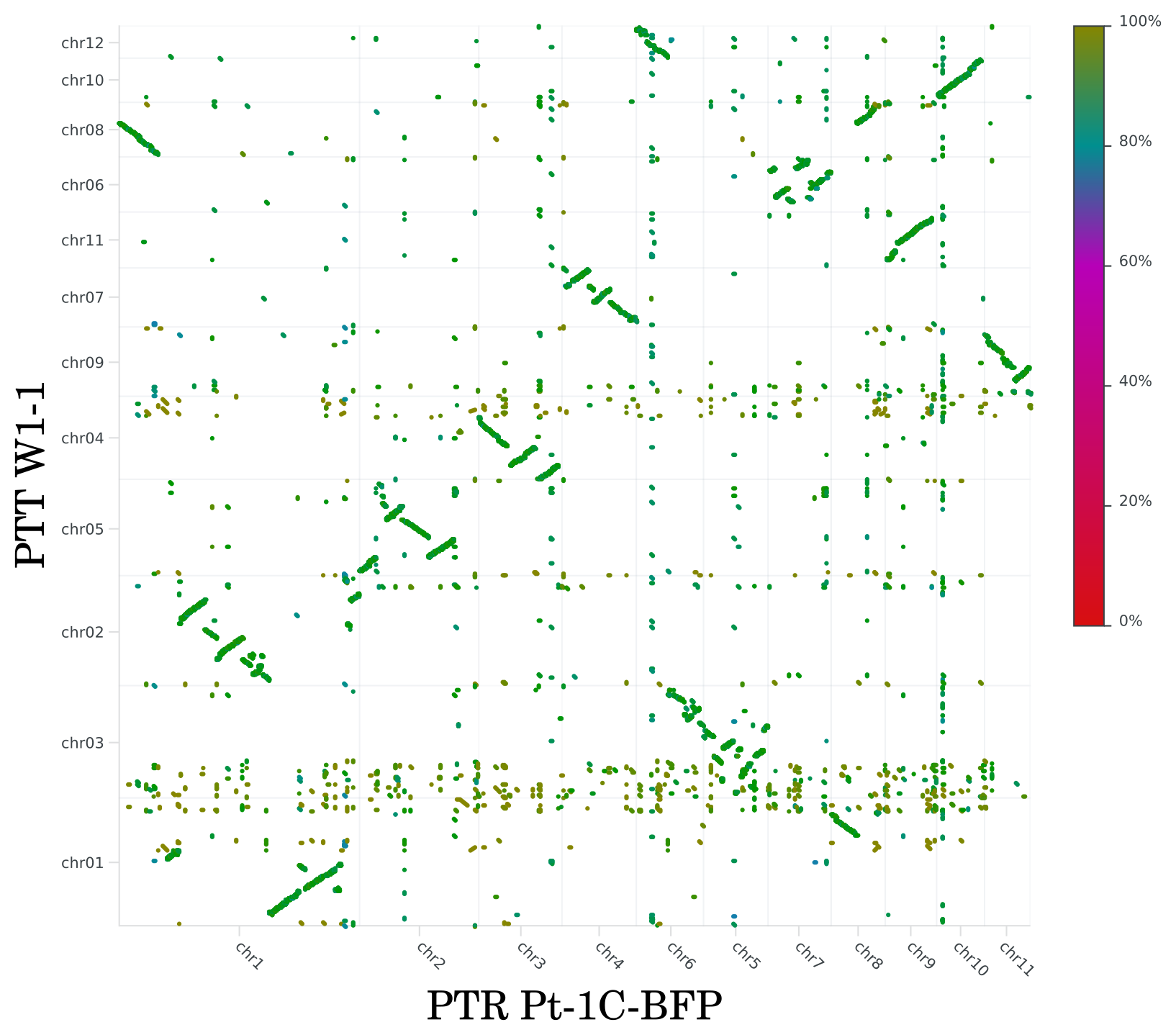

Supplement: Supplementary file 3 [file Data_Sheet_2.DOCX]
