# Supplementary material for: Transposable Element Genomic Fissuring in Pyrenophora teres Is Associated With Genome Expansion and Dynamics of Host–Pathogen Genetic Interactions
Source: Front Genet. 2018 Apr 18;9:130. doi: 10.3389/fgene.2018.00130 (PMC5915480; doi:10.3389/fgene.2018.00130)

**Supplementary Figure 3.** TE repeat family abundance in PTT W1-1 and PTM SG1 assemblies using a log-transformed y axis to show low abundance TE families in Figure 5.


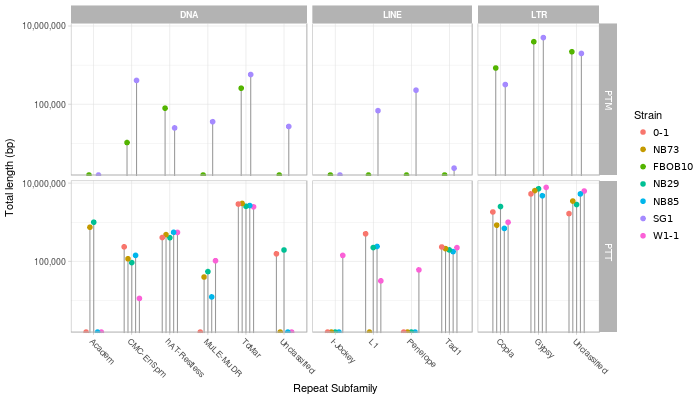

Supplement: Supplementary file 4 [file Data_Sheet_3.docx]
