# Supplementary figures and images for: Transposable Element Genomic Fissuring in Pyrenophora teres Is Associated With Genome Expansion and Dynamics of Host–Pathogen Genetic Interactions
Source: Front Genet. 2018 Apr 18;9:130. doi: 10.3389/fgene.2018.00130 (PMC5915480; doi:10.3389/fgene.2018.00130)

## Supplementary Figure 4. Bimodal G/C- read distributions in PTT and PTM.


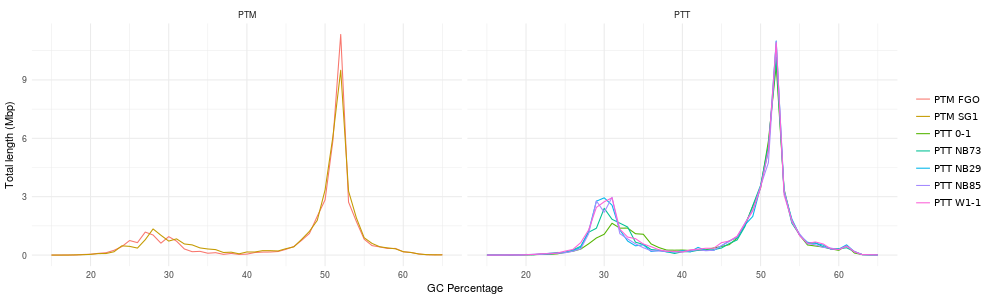

Supplement: Supplementary file 5 [file Data_Sheet_4.DOCX]
