# Supplementary material for: Transposable Element Genomic Fissuring in Pyrenophora teres Is Associated With Genome Expansion and Dynamics of Host–Pathogen Genetic Interactions
Source: Front Genet. 2018 Apr 18;9:130. doi: 10.3389/fgene.2018.00130 (PMC5915480; doi:10.3389/fgene.2018.00130)

## Supplementary Figure 5. Chromosomal fissuring between PTT W1-1 and PTM SG1 chromosome 10. GC% (blue) and AT% (green) indicate that the form-specific regions are low-GC, non-genic sequence. Fissuring sites in grey (requires image magnification) interrupt GC-equilibrated regions in the opposing strain.


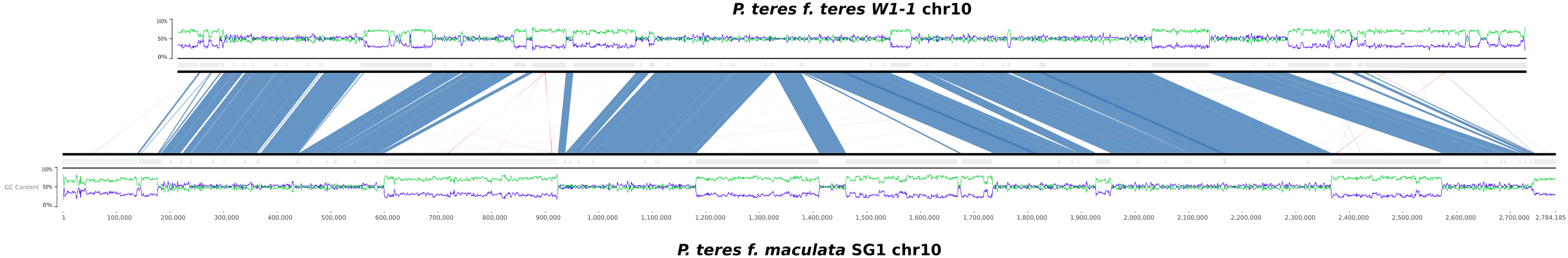

Supplement: Supplementary file 6 [file Data_Sheet_5.DOCX]
